# Seeds of authoritarian opposition: Far-right education politics in post-war Europe

**DOI:** 10.1177/1474904120947893

**Published:** 2020-08-19

**Authors:** Anja Giudici

**Affiliations:** Department of Politics and International Relations, University of Oxford, UK

**Keywords:** Politics of education, European education, far right, social movements, Neofascism

## Abstract

Since the 1980s, right-wing extremism, radicalism, and populism have emerged as transformative forces in European politics. This unexpected resurgence has triggered an interdisciplinary scholarly effort to refine our understanding of the far right. Educationalists, however, have largely been absent from this endeavour, leaving us unable to theorise and address the potential effects of the far right’s political and cultural growth on European education. This article aims to provide an empirically based conceptional groundwork for educational research on the far right. Drawing on archival research and content analysis of programmatic material produced by diverse and influential far-right organisations in France, (West) Germany, and Italy, I show that the post-war European far right disposes of the two essential features of a social movement: an action-oriented frame that reduces educational reforms to a common contentious theme, and a dense organisational network. The latter engages in institutional and contentious politics, as well as education. Theoretically, these findings suggest that, in the realm of education, the far right ought to be conceptualised as a social movement that seeks to influence education policy, and represents itself an educational actor. Addressing the far right’s multifaceted educational engagement thus requires a combined effort across European education research.

## Introduction

In the last few decades, the far right has moved from fringe status to mainstream player in European politics. A series of highly publicised electoral gains has granted far-right parties unprecedented access to political institutions, both at the level of the states and the European Union. But it is not only among voters that the far right seems to have lost its stigma. Far-right intellectuals’ access to the mainstream media has progressively established their ideas as a legitimate alternative in the societal debate (Mudde, 2016; Rydgren, 2018). In the meantime, far-right parties are increasingly being considered as partners for government coalitions, while their policy preferences have found their way into other parties’ programmes (Abou-Chadi and Krause, 2020; Mudde, 2019). In the twenty-first century, the far right has become part of Europe’s ‘political normalcy’ (Minkenberg, 2000: 170).

Far-right organisations are heterogenous. They range from neofascist collectives such as Casapound Italia to populist radical-right parties like the French Rassemblement national (RN), the former Front national (FN). But despite their organisational and programmatic differences, these organisations profess a common ideology (Carter, 2018; Mudde, 2000). The far right can thus be defined as an ensemble of actors sharing a set of distinctive ideological features. These include: a belief in authoritarianism; anti-democratic or anti-liberal attitudes; as well as an exclusionary or holistic understanding of nationalism (Carter, 2018). These features draw on a monistic understanding of society that fundamentally opposes liberalism and equality (Art, 2012; Carter, 2018; Mudde, 2010). Accordingly, analysts consider the resurgence of the far right ‘the most formidable new political challenge to liberal democracy in Western Europe and elsewhere’ (Betz and Johnson, 2004: 311).

In political science, history, and sociology, these developments have triggered a wave of new studies. This has led to a revision of classic understandings of far-right attitudes as the result of individual pathologies and a problematic upbringing (Mudde, 2010), such as in the case of Adorno et al.’s (1950) *Authoritarian Personality*. Far-right attitudes, the new scholarship argues, are by no means alien to Western post-war democracies. Support for the far right exists across time and Western electorates, and thus cannot be reduced solely to individual predispositions, voter anxiety, or economic change (Bale, 2017; Minkenberg, 2000; Mudde, 2010). Therefore, understanding the determinants and effects of the far right’s political presence requires a concerted effort to integrate this phenomenon into mainstream theories of societal and political change (Blee and Creasap, 2010; Mudde, 2000). As part of this effort, detailed knowledge has been produced on issues relating more closely to these disciplines’ core concerns. These involve: the motives and socio-demographic characteristics of far-right voters, including their education (e.g. Cavaille and Marshall, 2019); the far right’s ideology, organisation, and networks; as well as its policy preferences in selected areas such as immigration, and – to a lesser extent – welfare, gender and Europe (for recent overviews see Mudde, 2016, 2019).

However, so far, none of these disciplines has taken an interest in the far right’s education politics, ideas, or practices. Apart from a few UK and US-based exceptions (see The far right and education: literature review), neither have education scientists. Within continental European education in particular, the upsurge of the far right has been treated almost exclusively as a phenomenon in need of an educational solution. Thus, while a meaningful discussion has emerged on how education ought to react to attacks on liberalism and equality (e.g. Akbaba and Jeffrey, 2017; Riddle and Apple, 2019), virtually no study has engaged with far-right educational views or politics per se.

Bringing these views into the educational literature is increasingly relevant. The far right not only shows an ever-growing presence on the street and in the media, thus representing a potential influence for educational practices and debate, but its electoral gains also mean the movement has increased its representation in institutional politics, including the myriad of boards and committees shaping the decentralised policy field that constitutes education today. Our lack of systematic knowledge about how the far right approaches education, and to what aim, leaves us unable to assess whether and how it might have reshaped the educational debate and politics – as it has done in other policy areas (Mudde, 2019). Such knowledge also constitutes a precondition for educational scientists and professionals to articulate an informed stance towards the phenomenon (Apple, 2006). As argued by Mabel Berezin (2019: 357), concepts such as fascism or populism can serve as heuristic tools to ‘clarify our expectations of what we think a viable and inclusive democracy would be’, including in educational terms.

This paper provides key conceptual foundations for such a research programme. It tries to delineate the type of phenomenon the far right constitutes in education, and draws conclusions for promising theoretical approaches and avenues for research. Therefore, the paper primarily engages with issues of organisation and framing rather than with policy preferences and ideas. Indeed, to systematically address such preferences, as well as the dynamics behind their formation and dissemination, insight into the far right’s configuration and strategies is an essential prerequisite. This knowledge will also help us in identifying the channels through which far-right ideas might shape political decision-making and the societal (and academic) debate. The paper thus addresses two questions. Has the European far right shown any interest in or engagement with education in the post-war period? If yes, what does this engagement look like analytically, and what are the implications for how we ought to approach the far right in the realm of education?

To answer these questions, the paper relies on an interdisciplinary framework. More specifically, I review the recent literature on the far right in order to distil this phenomenon’s essential features (Defining the far right: essential features and internal variation). I then apply this definition to the educational literature in order to identify studies that, while carried out in isolation from the literature on the far right, have actually concerned themselves with far-right actors (The far right and education: literature review). Their findings, I argue, suggest that, when it comes to education, it might be beneficial to conceptualise the far right as a social movement rather than a partisan or intellectual phenomenon.

The second part of the paper assesses the appropriateness and analytical benefits of such a conceptual lens. I perform qualitative content analysis on a large database of programmatic documents (Methodology and data) produced by a selection of influential organisations representing the relevant cleavages within the post-war continental European far right, in France, Italy and (West) Germany. The analysis aims at establishing whether these organisations’ educational engagement displays the two essential features of a social movement, that is, whether they adopt an action-oriented frame that interprets education as a salient problem requiring political action (Far-right framing of education), and a dense organisational network that engages in institutional as well as contentious politics (Far-right educational networks).

I find the analysed organisations possess both these features. The continental European far right, therefore, not only views education (policy) as a salient issue, it also possesses the means for sustained engagement in this topic. This engagement involves both politics and education, which are considered either complementary or substitutive means to advance the far right’s struggle to change education policy, and, in turn, society and politics at large.

As argued in the conclusion, these findings highlight both the crucial contribution educational research could make to the literature on the far right, and the benefits our discipline could derive from considering the far-right case. They also have theoretical implications. Crucially, they suggest that our engagement with this topic cannot be limited to parties’ publicly declared preferences. The far right constitutes an international, multifaceted actor with a complex understanding of education politics. Its analysis requires an equally international, multifaceted, and cross-disciplinary effort. The concepts and theories developed by social movement research, this study suggests, provide a suitable common ground for such a combined research agenda.

## Defining the far right: essential features and internal variation

In the last few years, the literature on the far right has somewhat shifted its focus from voters to the far right itself. Departing from the classic understanding of far-right attitudes as individual pathologies, this literature defines the far right in terms of ideology (Carter, 2018; Mudde, 2000). Ideologies are characterised as ‘all-encompassing sets of ideas’ (Bale, 2017, 11) that supply actors with principles to interpret and judge social reality. They constitute the abstract framework upon which more volatile ideas and policy preferences concerning specific issues, such as education, are drawn (Berman, 2001; Mehta, 2011). At the same time, ideologies ‘link people who would not otherwise be linked’ (Berman, 2001, 105), thus providing a basis for party families and political communities. Defined as an ideology, therefore, the far right denotes a distinctive set of beliefs as well as a corresponding community of actors.

Different definitions of far-right ideology have been advanced (Carter, 2018; Mudde, 2019). In a recent contribution, Elisabeth Carter (2018), proposes a way out of this conceptual debate. Combining a comparison of the most eminent conceptualisations in the field with an empirical analysis of a large selection of far-right organisations, she whittles the ideology down to three essential features constituting a ‘minimal definition’ (Carter, 2018, 157) of far-right ideology.

The first, *authoritarianism*, is defined as a desire for a strictly ordered society where infringements are severely punished (Mudde, 2000). Underpinning this desire is an ideological appreciation of rigid social norms, discipline, and compliance (Carter, 2018). The second defining feature is *anti-liberalism* or -*democracy*. Anti-liberalism characterises the *radical* right, a term used to denote the far-right strand that, while opposing fundamental liberal tenets such as pluralism or equality does not reject democracy in itself, and complies with its procedures (Art, 2012; Carter, 2018; Mudde, 2010). Anti-democracy, on the other hand, qualifies the *extreme* right. This strand rejects democracy outright, even in its minimal definition as a system with regular free elections and guaranteed civil liberties. The third essential feature of far-right ideology is the belief that some social groups – mainly nations, but also genders, races, or religions – are transcendent and organic realities. With the assumption that humans are inherently unequal as its foundation (Carter, 2018; Mudde, 2019), this belief can lead to the kind of *exclusionary nationalism* that finds its expression in the xenophobic rhetoric and anti-immigration policies embraced by most contemporary far-right parties. It can also lead to a less exclusionary *holistic nationalism*, such as that promoted by Italian neofascists, which defines the nation in monistic terms and requires individuals to subordinate to its goals and will.

It may surprise that this definition does not include the buzzword populism. Indeed, scholars agree that populism, ideologically defined as ‘an appeal to “the people” against both the established structure of power and the dominant ideas and values of the society’ (Canovan, 1999: 3) is neither essential nor exclusive to far-right ideology. While championed by many contemporary figureheads, this attitude is actually very much alien to the elitist worldview of neofascists and new-right think tanks, where ‘the people’ ought to be led by a knowledgeable and morally sound elite, rather than the other way around (Bar-On, 2007; Carter, 2018; Mudde, 2009).

By drawing the boundaries of the far-right community based on ideology – instead of, for instance, more concrete and context-sensible policy preferences – it becomes possible to identify actors who have embodied this thought at different times and in different places, and explore how they relate to each other and their context (Carter, 2018; Mudde, 2000). This is especially advantageous since the literature finds the far right to constitute an inherently international, and European, phenomenon (Griffin, 2000; Mammone, 2015), whose representatives show some degree of programmatic and organisational variation.

On the one hand, the far right’s programme has evolved substantially since the 1950s. Von Beyme’s (1988) prominent three-wave systematisation proves a useful tool for capturing this evolution. Accordingly, the first wave consists of the neofascist organisations of the immediate post-war period, which recruited both personnel and programmes from interwar fascism. Starting in the 1960s, the second wave saw the foundation of mildly successful new parties which combined far-right ideology with populist and welfare-critical attitudes, such as the National Democratic Party of Germany (NPD). The crucial innovation in this period, however, came from New Right think tanks. They were founded by intellectuals convinced that a change of culture, of people’s hearts and minds, was the precondition for the far right acquiring political power. Therefore, they exited institutional politics, dedicating themselves to infiltrating the societal debate and rendering the far right’s programme more palatable to contemporary electorates. As part of their metapolitical struggle, they shifted the far-right programme from economic to cultural issues, replacing stigmatised ideas of corporatism, elitism, and racist nativism, with concepts such as ethnopluralism and identity (Bar-On, 2007; Capra Casadio, 2014; Griffin, 2000). In the third wave, starting in the 1980s, these ideas were implemented across Europe in the programmes of new, increasingly successful populist radical-right parties such as the Front National (Copsey, 2018; Rydgren, 2018). This development heralded what Cas Mudde has recently argued constitutes the fourth and latest wave. Starting at the beginning of the 21st century, it represents a phase in which far-right politics has gone mainstream, becoming ‘largely detached from the populist radical right parties’ (Mudde, 2019: 22).

On the other hand, within each of these waves, two further types of variation emerge. One relates to far-right representatives’ differing attitudes towards democracy. Indeed, subscribers of what analysts call the radical right, while criticising its liberal components, do not oppose a minimal definition of democracy. By forming parties that stand for elections, or engaging in the cultural debate, they also choose to defend their views within the legal and institutional scope of current democracies (Carter, 2018; Copsey, 2018; Mudde, 2010). This programme and strategy differ from extreme-right parties’, student militias’ or Neo-Nazi gangs’ revolutionary attitudes and outright opposition to democracy as a political means and end. A second crucial variation, then, is these actors’ different organisational and strategic set up. While the literature’s focus overwhelmingly lies on far-right parties, far-right ideology can also be embodied and promoted by other forms of actors, including intellectual think tanks as well as grassroots and subcultural societal organisations (Blee and Creasap, 2010; Castelli Gattinara and Pirro, 2019; Veugelers and Menard, 2018).

To sum up, the literature suggests that, to investigate what type of phenomenon the far right constitutes in the context of post-war European education politics, we can delimit the field based on ideology. At the same time, to be as comprehensive as possible, our analysis must consider the far right’s programmatic change, as well as varying organisational structures and attitudes towards democracy – both in its analytical (The far right and education: literature review) and methodological approaches (Methodology and data).

## The far right and education: literature review

Research on the far right and research on education show virtually no overlap. This is especially true for the English, German, French, Italian and Spanish language literature on Western Europe that has been considered here, together with English-language research on other parts of the world. Studies on the far right mention some parties putting education at the top of their agenda, for instance the German Republikaner (Minkenberg, 2001; Mudde, 2000), or new-right think tanks viewing education as a key lever in their metapolitical struggle (Bar-On, 2007; Capra Casadio, 2014). However, none of these studies have analysed these views in more detail.

On the other hand, educationalists who have engaged with post-war far-right actors have often treated them in isolation. This applies to the recent inquiries into German far-right think tanks’ views on upbringing and schooling collected by Andresen and Oelkers (2018), as well as the more extensive research on the 1980s English New Right (Ball, 1990; Chitty, 1989; Quicke, 1988) and Christian-nationalist, neoconservative, and white power movements in the US (Apple, 2006; Nickerson, 2012; Simi et al., 2016; Stewart, 2017). With one exception (Simi et al., 2016), these studies do not engage with the broader literature on the far right. However, both the English New Right and US Christian-nationalists belong to the far right according to our definition, making this research a precious point of departure for this investigation.

Taken together, these studies pinpoint two main commonalities between these actors. First, both groups display an extraordinary interest – US scholars speak of an ‘obsession’ (Stewart, 2017: ii) – in education. Second, they have not limited their struggle to change education policy to institutional politics, i.e. participation in parliaments and governments. Instead, the English New Right targeted the public debate as well as the programme of the Conservative party and government (Ball, 1990; Chitty, 1989; Quicke, 1988). Across the Atlantic, in the 1960s the US Christian-nationalist movement relied mainly on grassroots ‘kitchen table activism’ (Blee and Creasap, 2010: 274), with women taking their opposition to progressive education policies to the streets, the media and courts. In the 1970s, these efforts were complemented – but not substituted – by a concerted campaign to lobby established parties (Apple, 2006; Bates, 1991; Hixon, 1992; Nickerson, 2012; Stewart, 2017).

The far-right political community at large has been approached from different theoretical perspectives. Some scholars treat it as a party family (Mudde, 2000), others as a school of thought (Bar-On, 2007), and still others as a social movement (Castelli Gattinara and Pirro, 2019; Minkenberg, 2019; Veugelers and Menard, 2018). The aforementioned studies suggest that, when it comes to education, the US and UK far right might be best understood as a social movement, and thus as a form of political contestation where legislative action, grassroots mobilisation, and theoretical elaborations constitute complementary or substitutive means for promoting change.

Does this also hold for the continental European far right? It should be noted that, in the US and UK, the far right shows some peculiar organisational and programmatic traits. First, these countries’ majoritarian electoral systems discourage the formation of new political parties, obliging minoritarian ideologies to rely more heavily on bottom-up activism and lobbying (Blee and Creasap, 2010). Second, the neoliberal credo (Apple, 2006; Stewart, 2017) and the Christian fundamentalism (Hixon, 1992) found to underlie the UK and US far-right’s educational engagement are not as widespread in Continental Europe (Mudde, 2007). Moreover, the groups analysed so far represent typical second and third wave organisations engaged in a struggle against perceived cultural elites. These traits might enhance these organisations’ interest in education as well as their reliance on alternatives to institutional politics. Whether the continental European far right ought to be studied as a social movement, then, must first be established empirically.

The vast literature on social movements defines these as a specific form of political challenge. In addition to sharing a collective identity, as the far right does, movements ‘are involved in conflictual relations with clearly identified opponents’ and ‘are linked by dense informal networks’ (Della Porta and Diani, 2006: 21). For education research, movements are considered of particular interest because, as part of their political struggle, they often target formal education (Apple, 2006) as well as engage in educational activities themselves, for instance recruiting and training participants or trying to educate the larger public about their cause. As argued by Niesz et al. (2018: 3), ‘education is fundamental to social movements, and movements are fundamental to education’. According to this literature, then, to act as a social movement, a collective challenge needs to show two distinctive features.

First, social movements have to adopt action-oriented frames (Della Porta and Diani, 2006; Snow, 2004; Tarrow, 2011). Frames are schemata that guide how individuals perceive the world. As a sub-category, action-oriented frames show a distinctive agentive and contentious character: they politicise a topic and provide legitimation for collective action (Gamson, 1992; Snow, 2004). This means attributing selected events and experiences to clearly identified opponents, thus reducing them to corruptive ‘coalitions of interest’ (Della Porta and Diani, 2006: 21), rather than natural or divine responsibility, or incompetency. Framed as such, issues can be solved by human agency. Therefore, action-oriented frames supply individuals with a shared identity and legitimate collective action (Della Porta and Diani, 2006; Gamson, 1992; Snow, 2004).

Second, to engage in a sustained campaign for change and survive periods of low mobilisation, movements depend on ‘a dense social network and effective connective structures’(Tarrow, 2011: 16). Social movements typically neither adopt a centralised organisation nor do they control institutional power. Therefore, they rely on diverse organisations – parties, grassroots organisations, think tanks – that coalesce into more or less formalised networks. This enables them to combine tools of the extensive repertoire social movements have historically developed to engage in contentious – i.e. non-institutional – politics, such as demonstrations, petitions, lobbying, interactions with the media, or the establishment of their own channels of information (Tarrow, 2011).

The rest of the study thus asks the question: does the post-war continental European far right possess an action-oriented frame as well as a dense network and shared identity when engaging with education?

## Methodology and data

Considering the international orientation and internal heterogeneity of the far right, as well as the lack of standardised and comparable data on its various constituents (Mudde, 2016), this article presents a diachronic analysis of a carefully selected sample of organisations (see Case selection) based on original data (see Data and analysis).

### Case selection

Exploratory studies benefit from analysing cases that are particularly representative or influential for the phenomenon under scrutiny (Gerring, 2007). Therefore, my sampling aimed to select organisations considered to represent the far right’s internal variation, and at the same time to have played an influential role in shaping the movement. This meant, first, ensuring that the sample included extreme and radical organisations, and second, that it comprised parties, think tanks, and societal organisations. Since the European literature does not mention societal organisations dedicated to education, I tried to locate them empirically. I was also careful to add representatives of each of the three waves that have shaped far-right mobilisation up to the 2000s. The long timeframe allows consideration of actors’ dynamic and strategic nature by capturing how they adapt to institutional and contextual change (Mammone, 2015; Rydgren, 2005). I excluded the fourth and current wave, mainly because this is described as the phase in which far-right politics becomes detached from far-right actors, making it difficult to identify relevant actors. Sampling was also restricted to countries formally adhering to liberal-democratic tenets during the timeframe of the analysis, and thus authoritarian regimes (Spain, Portugal) as well as the Eastern Block were excluded.

To identify relevant organisations within each type, I relied on the extensive comparative scholarship on the European far right (Bar-On, 2007; Carter, 2005; Mudde, 2007; Norris and Ingelhart, 2019). The selection process was facilitated by the fact that, while the comprehensive mapping of the far right is contested, disagreement tends to be limited to borderline cases. On the other hand, there is a series of organisations whose relevance is universally recognised. The inclusion of these cases automatically fulfilled the third criterion: the consideration of multiple national contexts.

Because these relevant organisations are located in France, Italy and (West) Germany, the study focusses on these countries. Table 1 displays the main cases selected for analysis. For a comprehensive list that includes the organisations identified empirically, please refer to the online appendix.

**Table 1.** Main organisations.

**Organisation**	**Strand**	**Wave (origin)**	**Type**
**France**			
FEN (1960–67) & Occident (1964–68)	Extreme	1^st^	Student org.
GRECE (1969)	Radical	2^nd^	Think tank
Club de l’Horloge (1974)	Radical	2^nd^	Think tank
FN/RN (1972)	Radical	3^rd^	Party
**Italy**			
MSI/AN (1946–2009)	Radical	1^st^ / 3^rd^	Party
Nuova Destra (1970s)	Radical	2^nd^	Think tanks
**Federal Republic of Germany / Germany**			
NPD (1964)	Radical/extreme	2^nd^	Party
Neue Rechte (1980s)	Radical	2^nd^	Think tanks
Die Republikaner (1983)	Radical	3^rd^	Party

FEN: Fédération Étudiants Nationalistes; GRECE: Groupement de Recherche et d’Études pour la Civilisation Eu-ro-péenne; FN: Front national; RN: Rassemblement national; MSI: Movimento Sociale Italiano; AN: Alleanza Nazionale; NPD: Nationaldemokratische Partei Deutschlands

Note: For a comprehensive list of all the analysed organisations and sources please refer to the online appendix.

The initial sample includes what is widely acknowledged as the paradigmatic first-wave party, the Italian Movimento Sociale Italiano (MSI). Italian fascists profited from a political amnesty in 1946, which turned the country into a major node in the European far-right network (Mammone, 2015). The MSI thus became the longest-living neofascist party and an influential model for subsequent organisations, including the FN (Carter, 2005; Mammone, 2015; Mudde, 2019). In 1995, the MSI was re-named Alleanza Nazionale (AN) and voicing more moderate tones, it joined the national government.

The second wave is represented by the German Nationaldemokratische Partei Deutschlands (NPD) and other minor parties, as well as the think tanks and societal organisations that stepped in to renew the far-right programme in the 1960s, including the leading French Groupement de Recherche et d’Études pour la Civilisation Européenne (GRECE) (Bar-On, 2007; Veugelers and Menard, 2018). Student organisations such as the Fédération Étudiants Nationalistes (FEN) and Ordre Nouveau also offer an insight into extreme-right politics (Bale, 2017). While several organisations mentioned so far endured and adapted to the third wave, the acknowledged archetype of a third-wave radical-right populist party is the FN. As the first party to capitalise on new-right think tanks’ innovations, it became a model for the far right all over Europe (Copsey, 2018; Ignazi, 1997; Kitschelt, 1995; Rydgren, 2018).

### Data and analysis

This study relies on primary sources. More specifically, I assembled a large database of documents produced and issued by the selected organisations. This strategy was meant to ensure that the data represent these organisations’ officially sanctioned positions, rather than potentially marginal views expressed by individuals in essays or interviews (Blee and Creasap, 2010; Mudde, 2000).

Following the advice of ideology analysts, the database includes sources documenting both the ‘front stage’ and ‘back stage’ of political organisations (Mudde, 2000, 20; see also Bale, 2017; Pirro, 2018). Front-stage sources are documents targeting the general public, such as manifestos, reviews for large readerships, or parliamentary speeches. They give insight into the more polished and unified discourse that organisations use to attract supporters and convince the public of their positions, and are particularly useful for investigating the presence of collective action frames (Snow, 2004).

Back-stage sources, on the other hand, are produced for members and supporters only. They include newsletters, minutes of proceedings, and internal communication used to deliberate and disseminate policies within organisations. They thus provide information on connective structures and coordination between organisations, as well as on the logics behind specific framings and mobilisation strategies (Blee and Creasap, 2010; Mudde, 2000). These sources are more difficult to retrieve. Therefore, collection was limited to France and Italy where I had access to private archival holdings.

To identify relevant sources, I relied on the aforementioned literature on the European far right and on single-case studies (see online appendix). Data was mostly retrieved from libraries and archives in the selected organisations’ respective countries, where catalogues allowed me to identify further documents issued by these organisations and their publishing houses. I scanned these catalogues, archival holdings, reviews and newsletters systematically to identify two types of text unit. These are, first, books, articles, chapters, or statements delineating the organisations’ critique of the present world and the vision they want to achieve, and second, texts that either criticise the present schooling system or advance reform proposals. Within these text units, the extracts dealing with education served as coding units for the subsequent analysis.

The resulting body of texts was coded and interpreted systematically following the proceedings of qualitative content analysis. This means that inferences were drawn from the presence or absence of certain content characteristics, rather than their frequency (George, 2009). I coded the material thematically (Prior, 2014), registering passages discussing either: (a) the state of present education; (b) strategies to address this state. The body of text included in category (a) was used to assess the existence of action-oriented frames. This meant inquiring whether education and schooling are framed as contentious and agentive issues. I used texts in category (b) to systematically identify organisations involved in education politics, their connections and respective roles. Finally, I compared findings across organisations in order to identify common patterns characterising the far right as such. Less attention was paid to countries’ programmatic or organisational specificities.

## Far-right framing of education

The far right’s deep interest in education, this analysis suggests, is neither specific to the UK and US, nor does it only characterise culturally focussed third-wave organisations. Indeed, while sometimes other reasons are advanced to explain why contemporary societies do not align with what is supposed to be their organic, monistic and hierarchical order – such as the media, psychological or health problems – the causal role of schooling and education takes centre stage in the analysed organisations’ narratives. Revealing a strong belief in the power of education to shape society, they frame education as a salient grievance, pinpointing misguided education policies as main causes for the dire state of the present. These misguided policies are typically framed in contentious terms.

Indeed, the overwhelming majority of texts attributes these policies to the nefarious and deliberate choices of specific coalitions of interest. Definitions of these opponents change. However, the narratives always establish a connection between allegedly powerful international networks and local emissaries.

Hence, in the early post-war years, sources pinpoint the US as the main force behind European education reforms. Accordingly, by means of pedagogies drawing on ‘the modern and democratic way of thinking’, as disdainfully put by the MSI schooling expert Siena (in Istituto Nazionale di Studi Politici ed Economici [INSPE], 1960: 69), the US were trying to weaken Europe by subverting its natural authoritarian and hierarchical nature, thus ensuring their own supremacy.

From the 1960s onwards, the focus shifts to the left. In his public speeches, MSI leader Almirante regularly denounced ‘a plot for the Marxist takeover of schooling’ (in Almirante, Siena and Ruggiero, 1974: 4), steered by international communism together with local parties and teachers. Second- and third-wave think tanks and parties accused the new, post-1968 left of strategically occupying positions in educational administration and teaching, in order to use schools as ‘the continuation of the revolutionary struggle with other means’ (Gannat, 1993: 14). Reinventing the anti-capitalist programme pushed by Italian neofascists since the 1960s (e.g. Siena, 1972), the most recent frame pushed by the FN identifies yet another antagonist. Labelled the New Educational Multicultural World Order, this coalition supposedly includes corporations, international organisations such as UNESCO and the OECD as well as governments, local teachers’ unions, and, more recently, LGBT organisations (Frezza, 2017). Like its predecessors, the Order purportedly constitutes an ‘enterprise that aims to change attitudes on a global scale’ (Curtet, 1996: 6), and relies on schooling to uproot children from their native communities and render them defenceless against exploitation by global capitalists.

Taken together, the analysed texts reduce every thinkable dimension of contemporary education systems to these coalitions’ subversive aims. History and language curricula receive the most attention. The allegedly insufficient time dedicated to history in schools, biased depictions of historical facts (human rights, migration, fascism), as well as the relaxation of chronology and grand narratives are among the many dimensions of history curricula claimed to have been manipulated by the US’, the left’s, or capitalists’ wish to create a clean slate on which to construct a new, uprooted society (e.g. Club de l’Horloge, 1989; Rumpf, 1986). Similarly, innovations in language teaching such as the introduction of less grammar-focussed approaches, or the marginalisation of Latin are portrayed as deliberate efforts to ‘amputate national traditions’ and ‘flatten the brains of the new generations’, as MSI member of parliament Tripodi claimed (1962: 21).

However, curricula are not the only aspect of education reduced to political manipulation. Another prominent target of the analysed organisations’ critique is the expansion of secondary schooling. Reforms in this direction are considered part of a strategic plot to either stifle society by preventing the emergence of authority-inspiring leaders (e.g. Hoeres, 1993), or to create unemployed ‘professional dissenters and permanent revolutionaries’ (Curtet, 1995: 6). Similar accusations are brought against most other major post-war education reforms, as well as against minor issues such as school camps, which the FN women’s chapter described as a tool to undermine families’ influence on their children (Deleuze, 1991).

In short, the analysed organisations adopt a collective-action frame that depicts education as a salient grievance and allows them to effectively reduce the most disparate educational reforms to political contention, thus offering a rationale for action.

## Far-right educational networks

Does the far right, however, also have the means to engage in and sustain such action? The analysis presented in the following sections suggest it does. The Italian analysis (see Interlinked institutional and contentious educational politics) reveals how the far right built an education-dedicated network, and how this network could be activated to oppose educational reforms. The French analysis (see The complementarity of education and politics) describes the complimentary role played by politics and education as political means to promote change.

### Interlinked institutional and contentious educational politics

Until the collapse of the party system in 1994, the Italian far right was dominated by the MSI (Ignazi, 1998; Mammone, 2015). A rigidly organised neofascist party, the MSI had been represented in the Italian parliament since 1948. Believing, as expressed by Italian member of parliament de Marsanich, that ‘the holy war against communism is fought first and foremost in and with schooling’ (in INSPE, 1960: 65), the MSI parliamentary delegation regularly submitted propositions on education. Still, despite its monolithic structure and access to institutional politics, when it came to education, the MSI became just one of many nodes in a network of organisations mobilising streets and schools.

Since the 1960s, the Italian far right’s education politics have been formed through interactions between the party and non-party sectors. The latter was first represented by the INSPE, a think tank founded by far-right intellectuals in 1958. On the front stage, the INSPE described itself as non-partisan. However, the MSI entrusted the organisation with refining the party’s programme and forming future cadres (Tripodi, 1961). In 1960, the INSPE dedicated its annual conference to the issue of schooling. According to the event’s proceedings (INSPE, 1960), teachers, politicians, and academics from all over Italy assembled to discuss education and listen to talks by prominent intellectual supporters of Italy’s interwar Fascist regime. The event must have impressed the attending party leadership. Indeed, in the subsequent years, with the foundation of the Consulta Nazionale della Scuola and the Ufficio Scuola, the MSI also started reaching into the field. Founded in 1964, the Consulta periodically convened teachers and activists to discuss topical issues. The Ufficio Scuola, a permanent educational bureau created in 1969, was equipped with a page in the MSI’s official newspaper *Il Secolo d’Italia* (appearing weekly in the 1970s), as well as its own publishing house and news agency (both called Gnomes), which connected the bureau with school-based activists via phone and telegram newsletters (issued three times a week in 1975).

Indeed, from early on, the MSI had built a presence on the ground. The 1950s saw the creation of a youth organisation, Giovane Italia (rebranded Fronte della Gioventù in 1971) and the student organisation Fronte Universitario d’Azione Nazionale. Publications issued by their local, school- or university-based chapters testify to these organisations’ strong interest in education policy. They also display the effort dedicated to educating activists and connecting them through cross-school networks. Chapters such as Madri e Insegnanti per la Libertà degli Studi e la Difesa della Morale (Mothers and Teachers for the Freedom of Education and the Defence of Morals) fulfilled a similar role by providing women with a platform to discuss education and strategies ‘to undertake a work of persuasion, almost of secular mission’ (*Il Secolo d’Italia*, 1969: 3) within their gendered channels.

The central node in this network, however, was constituted of teachers. In the 1950s, they organised themselves in the Cisnal-Scuola syndicate. The organisation was rebranded Sindacato Sociale Scuola in 1977 (Scaramuzzino, 2018), at which point it was purported to include a million teachers (Stucovitz, 1976). The union not only represented its members’ interests, professing the belief that ‘no reform of schooling is possible without the firm and resolute conviction of teachers’ (Lozzi, 1973), it also convened discussions on education policy, organised teacher training, challenged education legislation in courts, and elaborated propositions for MSI representatives to submit to parliament. As testified by its newsletters and internal documents, the Cisnal-Scuola organised petitions and strikes to pressure the authorities. Despite being marginalised by democratic unions and governments, in some instances this strategy did allow them to enter negotiations.

That this network made use of connective structures enabling multi-site collective action is exemplified by the far right’s reaction to the Organi Collegali reform. Enacted between 1968 and 1974, the reform restructured Italian education governance by introducing decentralised boards staffed with elected parents, teachers, and students. Some boards had advisory capacity, others could deliberate issues such as the choice of schoolbooks, extra-curricular activities, or teachers’ performance reviews (Grimaldi and Serpieri, 2012). The far right greeted the reform with outrage. Not only did the idea of parents, teachers, and students deliberating on equal footing clash fundamentally with their ideal of ‘organic and hierarchical participation’ (*Il Secolo d’Italia*, 1974c: 2), the reform was also portrayed as a coup by communists, who had tricked the Government into opening new entry points for them on the ground.

Opposition materialised not only in parliament. As Cisnal-Scuola (1973a: 7, emphasis in the original) stated: ‘there is only one way to counteract these plans: to *participate en masse*’. Forms of participation varied, with youth chapters, the most extreme (and violent) part of the movement, taking their opposition to schools and the street. In their effort ‘to relaunch our values and proposing them as the only alternative again’ (Ruggiero, 1974), they relied heavily on the social movement action repertoire. In 1974, for instance, they organised ‘Weeks of struggle for schools’ involving public debates on education, demonstrations, and ‘propaganda interventions’ in several cities and schools (*Il Secolo d’Italia*, 1974a).

Teachers performed a more subtle, but no less crucial role. Like students, teachers were eligible for positions on the new governance bodies, and a steady stream of Cisnal-Scuola newsletters called for them to stand for election and ‘contribute to schooling’s transformation also from within its structures’ (Ciammaruconi, 1977: 1). Given teachers’ key position in between the party and local communities, they were assigned a further task: mobilising parents. Indeed, there are several indications that, in the 1970s, parents came to be increasingly valued as an activist base. For one, the increasing number of articles in the MSI’s *Secolo d’Italia* newspaper, trying to forge an identity for far-right parents as a community worrying about the safety of their children in schools dominated by leftist teachers and violent communist gangs, testifies to this appreciation. The value attributed to parents’ activism is also evidenced by the re-launch, in November 1973, of the Comitato Nazionale Genitori per i Problemi della Scuola e Famiglia (National Parents’ Committee for Problems of Schooling and the Family), rebranded Alleanza Nazionale Genitori Italiani (National Alliance of Italian Parents, ANGI) in 1975. Leadership was assigned to early neofascist activist and lawyer Eveno Arani. Via their newsletters, the Cisnal-Scuola and the MSI’s schooling bureau instructed teachers to report potentially sympathetic parents to the ANGI (Cisnal-Scuola, 1973b), whose declared mission was ‘to favour and coordinate the active participation of parents in the life of schooling’ (Commissione operativa, 1947).

On the front stage, organised parents concealed their ties with the MSI. Their uncoordinated appearance, however, was actually the result of strategic back-stage coordination. As ANGI leader Arani explained in a letter to MSI secretary Almirante, his organisation believed that most parents were inherently conservative regarding their children’s education and therefore agreed with the MSI’s educational programme. However, most parents were not political activists. Hence, the ANGI would profit from displaying a ‘non-partisan character’ (Arani, 1975), which it subsequently acquired. Instructions sent to local chapters indicate that, ‘by presenting themselves to the electorate without party labels [. . .] nationalist parents will be able to collect endorsements from wider sectors’ (Cisnal-Scuola, 1974). School election platforms of associated parents and teachers champion generic slogans such as: ‘A modern schooling where order and serenity reign’ (*Il Secolo d’Italia*, 1974d: 7). At the same time, back stage, the MSI’s schooling bureau and the ANGI provided parents with detailed instructions and support via a series of newsletters, a 24 hour hotline (*Il Secolo d’Italia*, 1974b), and a widely distributed booklet which, in addition to offering parents and teachers comprehensive legal and strategic advice, also includes verbatim arguments for them to use once elected – for instance, listing rigid Soviet school regulations they should quote to challenge students’ belief in communism (Bacci, 1975).

Due to the lack of centralised information, this and subsequent campaigns’ success is difficult to assess – even for the MSI itself. Still, it seems to have exceeded expectations given that MSI secretary Almirante personally expressed his gratitude to the ANGI for what ‘unexpectedly and almost miraculously has been realised and obtained in school elections’ (Almirante, 1975). This might explain why the strategy was subsequently expanded to other sectors.

In 1976, a debate on whether the Cisnal-Scuola’s affiliation with neofascism hurt its members’ interests split the union in two (Stucovitz, 1976). In subsequent years, however, the (much larger) component that continued to plead alliance to the MSI also started describing itself as ‘independent’ (Sindacato Sociale Scuola, 1979), before acquiring formal autonomy from the party in 1980. Yet the syndicate continued to collaborate intensively with the MSI leadership, and its ideological position did not change either. A recently published commemoration book calls Italy’s three Fascist education ministers the union’s ‘ideal and political points of reference’ (Scaramuzzino, 2018: 7).

Similarly, in 1978 the ANGI appointed a non-MSI member president. Nevertheless, the back-stage stream of communication with the MSI-leadership and schooling bureau remained intact. However, the organisation, now rebranded Associazione Nazionale delle Famiglie Italiane, also started collaborating with fundamentalist Catholic organisations, whose ideas on education and the family it considered akin to its own (Arani, 1978). Later rebranded Associazione Famiglia Domani, and directed by MSI financier Luigi Coda Nunziante, the organisation still exists today. As a member of the international Voice of the Family network, it continues to engage with education, for instance fighting against the ‘cultural revolution that aims to subvert the natural law by introducing gender theory in schools’(Associazione Famiglia Domani

### The complementarity of education and politics

Unlike its Italian counterpart prior to the FN’s first electoral successes in the early 1980s, the French far right did not have a party pulling the strings at the centre (Mammone, 2015). Still, the analysis presented here evidences that it developed a similarly complex engagement with education politics, based on a combination of institutional politics, contentious politics in schools, and educational activities.

The organisations that provided the breeding ground for the FN and the renowned French new-right think tanks were, in fact, educational organisations. Mobilised by the Algerian war, extremist second-wave organisations such as Ordre Nouveau or the Fédération des Étudiants Nationalistes (FEN) were mainly staffed by students, and focussed their activities on school and university politics. The FEN’s declared objective was ‘the eviction of Marxism from French universities and high schools’ (FEN, 1960). They considered the installation of ‘bases where nationalism rules’ (FEN, 1963) in educational institutions a priority because, in their elitist worldview, only a knowledgeable elite could provoke the ‘nationalist revolution’ (FEN, 1960) they yearned for.

This focus on education aligned with these groups’ larger approach to politics. According to Occident-leader Maurice Bardèche (1961: 189), contrary to the value-agnostic, educationally ineffective liberal state ‘[t]he object of the fascist state [. . .] is to form men according to a certain model’. At the same time, it followed a strategic rationale. The FEN leadership felt that, in the current democratic climate, people would never support a revolution if they perceived its advocates as a disruptive ‘united and visible front’ (FEN, 1963). By opting for a decentralised mode of action, however, the FEN leadership could not rely on organisational hierarchy and control to ensure militants on the ground embodied the organisations’ unified message and firm discipline.

They relied on education instead. Indeed, internal documents show FEN’s dedicated effort to devise a structured education system with a hierarchy of tutors and regular, carefully planned pedagogic settings to mould its activists. One such setting required activists to discuss articles on revolutionary struggles and extrapolate ‘the great fundamental laws of the revolutionary method’ (FEN, 1964). This group work ended with plenary presentations. The FEN’s Camps École, organised in the summers of the late 1960s and early 1970s, involved the future ‘elite revolutionary cadres’ in a combination of educational activities including sports, lectures on ‘pedagogy, propaganda, psychological guerrilla, and case study’, as well as rhetoric training to develop ‘the revolutionary reflexes, style, and intelligence that render one unassailable’ (FEN, 1973).

While renouncing the student organisations’ extremism, the organisations that succeeded them in the late 1960s borrowed their approach to education. New-right think tanks such as the Club de l’Horloge and GRECE frequently staged discussions on education. In 1976, these activities culminated in the foundation of the Groupe d’Étude pour une Nouvelle Éducation (GENE). Led by teachers, the GENE devoted itself to ‘elaborating a global educational project, as an integral part of a conception of life and the world’ (GENE, 1977: 2). A shiny review and a more sober newsletter testify to a sustained effort to develop a far-right position on topical issues, as well as elaborate theoretical foundations for a ‘new education’ based on a mixture of behavioural genetics, Europeanism and French pre-revolutionary thinking.

At the same time, the GRECE also saw itself as an educational institution. As part of its metapolitical Gramsci-inspired strategy, it worked to equip future public intellectuals with the knowledge and skills to occupy culturally influential positions in education and the media. As asserted by the GENE and GRECE leaderships, ‘the reconquest of political power goes through the (re)conquest of cultural power, whose foundations reside in education’ (Valclérieux, 1982: 4). Therefore, no election could be won ‘as long as we have not realised that a university seminar can be more important than a press conference or a party manifesto’ (De Benoist in GRECE, 1982: 19). The GRECE used tailored seminars and publications to disseminate their ideas among different publics, and organised scouting camps for youth (see Lamy, 2016).

Although it did not put education at the top of its front-stage agenda, the FN embraced a similar movement-like approach to what it called the ‘vast metapolitical field that is education and culture’ (*Français d’abord!*, 1995). The party’s appearance also supplied the French far right’s educationally engaged actors with the means of a coordinated network. This network was substantially reinforced after the left’s victory in the 1981 legislative elections. In response, FN leader Jean-Marie Le Pen proclaimed his so-called ‘water lily policy’ (Mayer and Sineau, 2002: 68), aimed at reinforcing the party’s presence in society via the foundation of professional and societal organisations. The fact that several of these new organisations concerned themselves with education attests to the issue’s importance on the party’s back stage. In addition to the youth chapter Front National de la Jeunesse (founded in 1973), these include among others: the Comités d’Action Républicaine (1982–1988) of FN ideologue Bruno Mégret; the women’s chapter Cercle National des Femmes d’Europe (CNFE) (1985 to 2000s), led by early FN activist and member of the European Parliament Martine Lehideux; and the Cercle National Education Nationale (CNEN). Rebranded Mouvement Éducation Nationale (MEN) in 1995, the latter claimed to have more than 2000 members and sympathisers among teachers, local chapters in 41 *départements*, as well as links with selected teacher unions (*Français d’abord!*, 1995).

While they did not possess a centralised structure, together these organisations formed a tightly knit and diversified network. As shown from conference proceedings, their members and representatives of the new-right think tanks participated in each other’s activities, producing statements and publications that then fed into the FN’s programmes and speeches. This type of action was intended to help politicians redesign the education system in the long term. At the same time, however, these organisations also joined forces in order to organise stakeholders on the ground. As the leader of the Comités d’Action Républicaine, Mégret, indicated to the women’s chapter, CNFE: ‘There are not only elections [. . .] there is also and above all a battle of minds, which is the one you are leading’ (CNFE, 1987). Therefore, parents, students and teachers were asked to exploit the current structures to transform education and the educational debate from the bottom up, by standing for school elections or voicing their educational ideas in public. Mothers’ engagement was especially solicited. They were considered to possess a particular ‘tenacity, lucidity and courage to fight for their own children [. . .] and for the memory of whom gave them life’ (Brissaud, 1991).

The organisations’ newsletters and events tried to establish the community needed to engage in this fight. Together with the FN’s weekly *National Hebdo* newspaper, they regularly denounced reforms, schoolbooks, or teacher behaviour that allegedly discriminated against right-wing parents and students, for instance by praising human rights or describing France as a multicultural country. These outlets also provided stakeholders with a platform where they could connect and exchange their experiences. They advised activists on how to organise demonstrations and interact with the authorities and media, offering pre-written letter templates and media releases. As in Italy, these suggestions are revelatory of the far right’s belief in parents’ inherently conservative educational attitudes. Therefore, they instructed parents and teachers not to disclose their party affiliation, but instead ‘discreetly but insidiously slip our ideas in occasionally and come back to these topics regularly [. . .]. Since they are full of common sense, it will be difficult to contradict you’ (CNFE, 1989a).

In the analysed publications, not only legislation and social movement tools, but also education itself is described as a means for societal and political change. These organisations regularly organised events aimed at educating activists and future cadres, such as the yearly FN Summer University. Other settings targeted the next generations at large. By participating in the disciplined outdoors activity of a month-long scouting camp delivered by the FN veterans’ chapter, parents were promised in the 1990s that their children would learn ‘what schools no longer teach them’ (*Français d’abord!*, 1996).

Parents themselves, however, were also asked to play their part. In addition to protesting nefarious education reforms politically, they should correct them educationally. Therefore, the CNFE’s newsletter recommended its (female) readers ‘to personally supervise your children’s civic education and to set the record straight when they come home from school’ (Payet, 1986). This and other outlets mention publishing houses producing ideologically aligned materials (for example, Fédération Internationale pour la Défense des Valeurs Fondamentales, France valeurs, and Éditions SDP) – that ‘will give parents clear guidance on how to teach their little ones the invariable truths’ (CNFE, 1989b), thus testifying to a somewhat concerted educational effort. Only by combining institutional and contentious politics with educational action, the FN’s newsletter declared in 1995, would the far right be able ‘to play the national card’ when a crisis was to finally hit the schooling system, and thus transform education – and society – for good (*Français d’abord!*, 1995). In this novel society, education was to play a crucial role. As expressed by FN president Jean-Marie Le Pen (1984), the prime purpose of his envisioned government was to firmly regulate individual behaviour for the sake of society and ‘to develop the good civilised’, which implied ‘the creation of a whole series of educational mechanisms’ (83–84).

## Discussion and conclusion

In her study of 1960s neoconservatism, Michelle Nickerson (2012: 4) argues that women’s grassroots activism not only changed the nascent US right, but also ‘raises questions about how women shaped political history through the minds of schoolchildren’ by systematically undermining progressive education reforms. These questions, she admits, are difficult to answer. Like hers, this study cannot provide insight into the effects of far-right activism on education policy, debates, or practices. What it does show, however, is that to address such effects, a focus limited to institutional politics, to far-right parties’ participation in elections, parliaments, and government, only reveals part of the picture.

This contribution asked how education researchers can analytically engage with the post-war far right and, more specifically, whether its Continental European strand can be studied as a social movement. Investigating diverse and influential far-right organisations in France, (West) Germany, and Italy, the study finds that the literature’s neglect for this topic is empirically unjustified. The far right does concern itself with education, and in doing so, it has developed the two features social movement scholars pinpoint as prerequisites for collective mobilisation and action.

First, the analysed organisations identify education as a highly salient issue, and one of the main drivers behind societal corruption. This is considered no accident, but the result of education policy being co-opted by particular coalitions of interest – the US, the left, and global capitalism. This allows the far right to reduce the disparate landscape of post-war educational reforms – from language teaching to school camps – to one educationally focussed collective action frame, providing militants with a rationale for action. Second, these organisations’ approaches are based on the belief that only a combination of institutional and contentious politics can lead to political and educational change, and that such change has to be underscored by both political and educational action. Therefore, the far right has developed a dense network of dedicated political and educational organisations that connect stakeholders in parliaments, intellectual think tanks, schools, and family homes.

Contemporary developments suggest that the far right has not changed its approach in what analysts call the fourth wave of far-right politics (Mudde, 2019). For instance, the FN, now Rassemblement National, has recently revived its chapters for teachers (Collectif Racine), students (Collective Marianne), and the youth (Génération Nation). In the last few years across Europe, organisations like these and their grassroots counterparts have joined religious fundamentalists in taking to the streets to protest educational issues, most prominently the teaching of gender (see, for example, Hoffmann, 2017; Khemilat, 2018). Dedicated websites continue to supply parents and activists with detailed information on supposedly dangerous educational projects and schoolbooks, and on how to oppose them politically and educationally. There have also been attempts to build more institutionalised forms of education. Prominent far-right spokespersons like Marion Maréchal and Steve Bannon have deliberately withdrawn from institutional politics in order to dedicate themselves to educating a new intellectual elite. The movement’s investment in the case of home-schooling (e.g. Daudet, 2019; Sommerfeld, 2019) – described by identitarians as a way to escape ‘the lethal cocktail the state administers its children’(Identità Europea, 2015) – provides further testimony to the continuing value of education in the far right’s political struggle.

Considering its limited geographic and thematic horizon, this analysis can only provide an initial insight into the post-war far right’s engagement with education. Furthermore, its analytical focus on organisations’ and networks’ commonalities neglects their changing and varying nature, while the focus on these networks as a whole overlooks the independent role played by its single components – including the increasingly influential far-right opposition (and government) parties that have risen across Europe. Still, the analysis highlights the multi-faceted nature of the far right’s education politics, the understanding of which necessitates an equally multi-faceted effort across the discipline of education. The concluding paragraphs draw on social movement theory to outline three avenues for research in the hope of encouraging European education research to engage with this topic.

First, while the analysis shows the far right’s opposition to almost every aspect of post-war education systems, we still lack knowledge about what it wants to replace them with. Education is a value-laden issue and an area where different visions of society are negotiated (Apple, 2006). A systematic study of how the far right’s educational ideas and policy preferences have diverged and converged, as well as how they travel within and outside the movement, thus promises crucial insight for research on the far right.

This type of research also promises important results in the field of education. Political ideologies contribute to shaping educational ideas and policies (Ansell and Lindvall, 2013; Apple, 2006; Mehta, 2013). At the same time, social movements are found to have dramatic political (Tarrow, 2011) and cultural impact (Amenta and Polletta, 2019), including upon education (Apple, 2006; Niesz et al., 2018). However, the tendency especially of European education research to focus on progressive actors (Niesz et al., 2018) – an inclination shared by movement research more generally (Blee and Creasap, 2010) – creates important gaps in our theoretical knowledge of how political ideologies affect, and are affected by, the politics of education.

This is especially true in Europe today, where the far right constitutes a growing political force which has set out to transform both national politics and the European community, and whose vision of Europe has increasingly gained traction (Vasilopoulou, 2011). Its views are crucial if, as recently called for by Seddon and Niemeyer (2018: 762) in this journal, we aim to better integrate understandings of ‘Europeanisation outside of Brussels’ into European education research. This study suggests that such research might profit from harnessing the analytical tools developed by the social movement literature to theorise how movements produce knowledge and frames, and use these resources to promote change.

Second, while focussing on their shared features, this study also discloses variance in networks’ configuration and action repertoires. For instance, the electoral affirmation of the FN in France or the decentralisation of education governance in Italy affected their respective networks. This raises questions about the determinants and consequences of the strategies chosen by the far right to tackle education’s notoriously dispersed authority structure (Ball, 1990). We might also ask whether these strategies differ from those of progressive movements, and thus how the far right’s organisation, activities and impact interact with its institutional and political context, as well as ideology. The social movement literature has developed sophisticated frameworks to theorise movements’ behaviour and impact relying on their organisational features, ability to emotionally activate participants and frame the debate, as well as the political and institutional opportunities and constraints determining their access to institutional politics (Della Porta and Diani, 2006; Tarrow, 2011). Insights from comparative analysis harnessing ideological, institutional, and geographical variance – and including a focus on different parts of Europe – would be especially valuable for applying these frameworks in education.

Third, this study corroborates Niesz et al.’s (2018: 2) claim that ‘movements themselves are educators’. Indeed, a large part of social movements’ activities consist of the production and dissemination of knowledge, skills and identities among activists and future cadres, as well as the public at large (Niesz et al., 2018; Simi et al., 2016). Sociological research also suggests parenting plays a crucial role in the survival and spread of social movements, with ethnographers showing far-right families to understand their parenting as activism and to engage in specific parenting practices, for instance when celebrating rituals (Simi et al., 2016; Veugelers, 2011). Whether and how these and other educational practices are actually shaped by ideology, as well as how educationally and politically effective they are, remains open to analysis.

Answers to these questions might not only enrich our understanding of how educational beliefs and practices form. Scholars of the far right have started calling for knowledge on ‘why people join, how they are socialized into members, and how the party picks and trains its cadres’ (Mudde, 2016: 13; see also Art, 2012; Miller-Idriss and Pilkington, 2017; Veugelers and Menard, 2018). Educationalists are ideally equipped to shed light on such processes.

## Supplemental Material

farright_eerj_appendix_200715 – Supplemental material for Seeds of authoritarian opposition: Far-right education politics in post-war Europe Click here for additional data file. Supplemental material, farright_eerj_appendix_200715 for Seeds of authoritarian opposition: Far-right education politics in post-war Europe by Anja Giudici in European Educational Research Journal
