# Supplementary material for: Seeds of authoritarian opposition: Far-right education politics in post-war Europe
Source: Eur Educ Res J. 2020 Aug 19;20(2):121–42. doi: 10.1177/1474904120947893 (PMC7948513; doi:10.1177/1474904120947893)
Supplement: farright_eerj_appendix_200715 – Supplemental material for Seeds of authoritarian opposition: Far-right education politics in post-war Europe [file farright_eerj_appendix_200715.pdf]

Online appendix for:
Seeds of authoritarian opposition: far-right education politics in post-war Europe

Anja Giudici, University of Oxford, anja.giudici@politics.ox.ac.uk

15th July 2020

List of Tables

		Page
1	Organisations & sources for France – parties	7
2	Organisations & sources for France – think tanks & societal organisations	8
3	Organisations & sources for Italy – parties (a)	9
4	Organisations & sources for Italy – parties (b)	10
5	Organisations & sources for Italy – think tanks & societal organisations	11
6	Organisations & sources for Germany – parties	12
7	Organisations & sources for Germany – think tanks & societal organisations	13

Introduction

This online appendix complements the paper "Seeds of authoritarian opposition". It provides more detailed information on the nature and location of the data underlying the analysis as well as a comprehensive list of the analysed sources. The primary aim of data collection was to provide systematic documentation on the far right's approach to education policy in post-war Continental European democracies. However, in the future, this type of data might also be used to study this ideology and movement's ideas and policy preferences regarding selected educational topics. Such studies are lacking in the European literature so far. Therefore, this Appendix is not only meant to increase the transparency of the analysis presented in the paper, but also aims to provide future research in the field with a systematic overview of potentially relevant organisations (section 1) as well as studies and sources relating to these organisations (section 2). The tables included at the end of the Appendix provide a comprehensive list of potentially interesting sources, grouped according to the criteria introduced in the first two sections.

1 Description of cases

The far right is defined here as an ideology based on authoritarianism, a holistic or exclusivist understanding of nationalism, as well as either anti-liberal or anti-democratic attitudes (Carter, 2018). Organisations subscribing to these tenets, constitute the potential universe of cases for studies on the far right's policy and politics.

According to the (extensive) literature on the European far right, these organisations show some characteristic variation. Given this study's exploratory aim, following Gerring (2007), data collection focused on a selection of cases that represent this variation while at the same time also play an influential role in shaping the European far right at large. Based on the literature in the field, four sources of variation were considered:

Internationalism After 1945, the European far right found itself marginalised. With parties and governments within their countries denying cooperation, far-right actors sought allies across national borders. Historians show that, as a result, the post-war far right has developed into a decisively international phenomenon. The movement's ideas and strategies have been shaped by international cooperation, as well as copying and lending mechanisms, with some organisations constituting hugely influential models subsequently emulated all over Europe (and beyond) (Albanese & Del Hierro, 2014; Griffin, 2000; Mammone, 2015; Rydgren, 2005).

Post-war evolution In the post-war period, the far right has evolved, showing distinctive programmatic and strategic characteristics in what analysts call its first- (1945-early 1960s), second- (1960s to 1980s), third- (1980s-2000s) (Mudde & Rovira Kaltwasser, 2015; von Beyme, 1988), as well as fourth waves (after 2000s) (Mudde, 2019).

Programmatic Scholars also distinguish a radical and extreme strand of the far right (e.g., Art, 2012; Carter, 2018; Copsey, 2018; Mudde, 2010). Organisations constituting the radical right subscribe to an ideology that combines far-right authoritarianism and an holistic or exclusivist understanding of nationalism with anti-liberalism. Hence, while they oppose fundamental liberal tenets such as equality and individual rights, they do not reject democracy as such – at least in its minimal definition as a system with regular free elections and guaranteed civil liberties. These organisations also generally comply with democratic procedures in their own organisation and strategies, for instance constituting parties that participate in elections. On the other hand, the extreme right is both anti-liberal and anti-democratic. Organisations constituting this part of the movement reject democracy as a principle of government, and often also refuse to comply with democratic rules to advance their political goals, opting for violence and terrorism instead.

Organisation Far-right ideology is not only embodied by political parties. Indeed, several studies highlight the importance of the non-party sector for advancing the far right's agenda. They pinpoint societal/grassroots organisations and think tanks as particularly important sources of far-right activism, and a crucial complement to parties' electoral and legislative politics (Bar-On, 2007; Blee & Creasap, 2010; Castelli Gattinara & Pirro, 2019; Veugelers & Menard, 2018).

Therefore, this study includes influential organisations that, taken together, represent the European far right's characteristic *international scope* (multiple countries); *evolution in time* (first-, second-, and third wave); *programmatic variance* (extreme and radical right); as well as *organisational variance* (parties, think tanks, and societal organisations).

Data gathering was limited to the period from the 1950s to the 2000s, and thus excludes the current, fourth wave of far-right politics. The latter is characterised by the mainstreaming of far-right politics, with parties and organisations across the political spectrum adopting ideas and policy preferences originally developed by the far right (Abou-Chadi & Krause, 2019; Krause & Giebler, 2019; Mudde, 2019). While it would be interesting to expand data collection to the current period of time, arguably, before we can trace whether and how far-right educational ideas travel beyond the movement, we first have to identify these ideas.

Different definitions of the far right and related concepts exist. Consequently, the delimitation and mapping of the empirical field is contested. However, disagreement tends to be limited to some borderline cases. On the other hand, there are a number of organisations whose influence and representativity is undisputed in the comparative literature, and which are included in all prominent categorisations of the far right – regardless of the specificities of their underlying definitions (Bar-On, 2007; Carter, 2005; Kitschelt, 1995; Mammone, Godin & Jenkins, 2012; Mudde, 2007; Norris & Ingelhart, 2019; Zaslove, 2009). Only cases where such unanimity exists were considered for analysis. The study thus excludes organisations whose status is contested or shifted during the analysed period.[1]

Among the uncontested organisations, I selected those the literature considers most influential in shaping the ideology and strategy of the aforementioned strands, as well as the European movement at large. The resulting sample included selected think tanks and parties in Italy, France, and Germany. I thus focussed my research on these three countries, where I also tried to empirically locate societal organisations dedicated to education, since information on this type of organisation was lacking in existing literature. A comprehensive list of the analysed organisations can be found Tables 1 to 7. For more detailed information on their significance and trajectory please refer to the paper.

2 Data

This study explores the politics and ideas of the organised far right. In terms of data, it relies on original documents officially issued by the selected organisations. Compared with potential alternatives, this strategy provides two analytical benefits (Art, 2012; Bale, 2017; Mammone, 2015; Mudde, 2000). First, it allows capturing these organisations' official and supposedly consensual opinion, rather than having to rely on retrospective, individual, and potentially deviant views expressed by members in interview situations. Second, it considers far-right organisations as actors in their own right, and does not reconstruct their activities or strategies based on what experts or their constituents think about them.

1. Examples of contested or shifting organisations are the Swiss Schweizerische Volkspartei, the Italian Forza Italia and Lega, or the Austrian Freiheitliche Partei Österreichs.

Following the advice of researchers of political ideologies, the analysis combines documents drafted for different means and audiences (Mair & Mudde, 1998; Mudde, 2000; Pirro, 2018). More specifically, I gathered sources documenting both the front-stage and back-stage of political organisations.

As outlined in more detail in the paper, *front-stage sources* are documents drafted with a wider public in mind. They include the more polished message an organisations wants to communicate to potential sympathisers and the public at large. They thus constitute a valuable source for assessing the presence or absence of action-oriented frames regarding a specific topic (Snow, 2004). Front-stage type of sources that have been collected for this analysis include:

* organisations' official daily newspapers or weekly magazines for the larger public;
* books written for non-specialised audiences;
* parliamentary speeches;
* organisations' manifestoes and official programmes;
* other types of propaganda material: flyers, brochures, etc.

Back-stage sources address an organisations' sympathisers, activists, and leaderships. They serve the purpose of internal communication and coordination, and thus are often more specific and less polished than documents aiming at the larger public. They provide insight into how organisations coordinate and communicate with other players, as well as into the strategic and programmatic considerations feeding into their front-stage communication. This type of documents is more difficult to retrieve. Unlike communication directed at the general public, organisations' internal communication is often stored in specialised or private archives. Therefore, collection was limited to Italy and France, where the most influential organisations are located and I could secure access to such archives and libraries. Back-stage sources on which the analysis is based include:

* newsletters for members and activists;
* more specialised literature issued for information and education purposes;
* circulars and internal communication between officials representing the organisation.

The first strategy I used to identify relevant sources was an extensive review of the case studies produced for the selected organisations and countries. Special attention was payed to the sources on which these studies are based. Relevant case studies include:

France: Almeida (2019); Bale (2017); Bar-On (2007); Birenbaum (1992a, 1992b); Camus (2015); Capra Casadio (2014); Mammone (2015); Mayer (2018); Seidel (1986);

Germany: Backes (2018); Brauner-Orthen (2001); Funke (2009); Gress, Jaschke and Schönekäs (1990); Minkenberg (1992); Mudde (2000);

Italy: Albanese and Del Hierro (2014); Bozzi Sentieri (2007); Capra Casadio (2014); Ferraresi (1984); Froio, Castelli Gattinara, Bulli and Albanese (2020); Griffin (1996); Ignazi (1998); Mammone (2015); Tarchi (2003); Veugelers (2011).

To complete the list of potentially relevant sources, I relied on library and archival catalogues, which allowed me to identify further documents issued by the selected organisations and their publishing houses. Researchers and archivists in the three countries as well as provided me with additional knowledge on the availability of data, as did some (former) activists. As shown in Tables 1 to 6, data was mostly retrieved from libraries and archives in the selected organisations' respective countries, including (the abbreviations in brackets are used in the tables at the end of the Appendix):

France

* Assemblée nationale, Service de la Bibliothèque et des Archives, Paris (AN-SBA);
* Bibliothèque Nationale de France, Paris (BNF);
* Les archives d'histoire contemporaine, Fond Etudiants Nationalistes, SciencesPo, Paris (Chsp-FEN);
* SciencesPo Bibliothèque, Paris (SPB).

Italy

* Biblioteca Nazionale Centrale, Firenze (BNC);
* Fondazione Ugo Spirito e Renzo De Felice (FUS).

Germany

* Deutsche Nationalbibliothek, Frankfurt (DNB).

Some data has also been digitalised and is available online. This includes party manifestoes, which have been digitalised for major European and French research projects, including:

* the Manifesto Project (MP): manifesto-project.wzb.eu, see Volkens et al. (2017);
* the Political Documents Archive (polidoc): polidoc.net, see Benoit, Bräuninger and Debus (2009) as well as Gross and Debus (2018);
* the Archives électorales du CEVIPOF Sciences Po (CEVIPOF): archive.org/details/archiveselectoralesducevipof.

In addition, most national and some regional parliamentary services have digitalised speeches and other parliamentary documents.[2] Lastly, some more recent material and communication aimed at a larger public can be found on the organisations' respective websites.

To complete this Appendix, Tables 1 to 7 list the organisations and sources considered for this study. The presentation is organised according to the aforementioned criteria: national context (France, Italy, Germany); type of organisation (Type: party, think tank, societal org.); programmatic orientation (Strand: Radical Right RR or Extreme Right ER); wave (Wave: 1st, 2nd, or 3rd); as well as, for sources, front- and back-stage (Reach: Front or Back).

For further information as well as access to the database please contact Anja Giudici, anja.giudici@politics.ox.ac.uk.

2. For instance Italy (archivio.camera.it) and France (archives.assemblee-nationale.fr).

Table 1: Organisations & sources for France – parties

Org.	Type	Strand, Wave	Sources description: *title*, dates of publication (dates analysed)	Reach	Location
**Front National (1972-...)**	Party	RR, 3rd	Weekly newspaper: *National Hebdo*, 1984-2008 (1984-85; 1987-91; 1995-96)	Front	BNF
			Monthly theoretical review: *Identité*, 1989-1996 (1989-96, issue 1993/19 dedicated to schooling)	Front	SPB
			Party manifestoes, 1972-... (1972-2012)	Front	MP & polidoc
			Speeches & interventions on education in the national parliament (1986-88; 2012-19)	Front	AN-SBA
			Manifesto books & official education literature: J.-M. Le Pen, 1984, *Les Français d'abord*; B. Gollnisch, 1985, *Une âme pour la France*; J.-M. Le Pen, 1985, *Pour La France*; Institut de Formation nationale, *Militer au Front*, 1991; J.-M. Le Pen, 1991, *Le Pen 90*; J.-M. Le Pen, 1995, *Le Contrat Pour La France avec les Français*; M. Le Pen, 2012, *Pour que vive la France*	Front	BNF, SPB
			Newsletters: *La lettre de Jean Marie Le Pen*, 1985-1990 (1985-1990); *Esprit d'entreprise* (1989); *Français d'abord!*, 1994-2008 (1994-2000, 2005-2008); *Nation Presse* 2009-15 (2011)	Back	BNF
**Cercle National Femmes d'Europe (1985-2000s)**	Party org. FN-♀	RR, 3rd	Newsletter: *Cercle National Femmes d'Europe*, 1985-2003 (1985-2003)	Back	BNF
**Front National Jeunesse**	Party org. FN-youth	RR, 3rd	Newsletters: *Fair Front!* (in *La lettre de JMLP*); *Agir pour faire front*, 1995-2007 (1995-98; 2000-01)	Back	BNF
**Mouvement Éducation Nationale**	Party org. FN-teachers	RR, 3rd	Manifesto book: O. Pichon, 1991, *Pour une instruction nationale*; articles: in *National Hebdo* & *Identité*	Front	BNF, SPB
			Articles: in *Français d'abord!*	Back	
**Renouveau Étudiant (1990-2000)**	Party org. FN-students	RR, 3rd	Semestral review: *Offensive pour une Nouvelle Université* (1997-98)	Front	BNF
**Mouvement National Républicain (1999-...)**	Party	RR, 3rd	Manifestoes & official education statements	Front	www.m-n-r.fr

Table 2: Organisations & sources for France – think tanks & societal organisations

Org.	Type	Strand, Wave	Sources description: *title*, dates of publication (dates analysed)	Reach	Location
**Fédération des Étudiants Nationalistes (1960-67)**	Student org.	ER, 2nd	Monthly magazine: *Cahiers Universitaires*, 1961-70 (1961-69)	Front	BNF
			Internal newsletter: *FEN-presse* (1964-66)	Back	Chsp-FEN env. 1, dossiers 1-8
**Europe Action (1963-66)**	Student org.	ER, 2nd	Monthly magazine for members: *Europe Action*, 1963-66 (1963-66)	Back	Chsp-FEN env. 3
			Archival files: internal communication re. organisation, schooling, strategy, including internal newsletter *Militant* (1969-70)	Back	Chsp-FEN env. 2, dossiers 1-3
**Occident (1964-68)**	Student org.	ER, 2nd	Book: M. Bardèche, 1961, *Qu'est-ce que le Fascisme?*	Front	BNF
			Monthly reviews: *Voix de l'Occident* (1962); *Occident Université*, 1965-66 (1965); *Défense de l'occident* 1952-83 (1968-83)	Back	BNF
**Groupement de recherche et d'études pour la civilisation européenne (1968-...)**	Think tank	RR, 2nd	Monthly magazine: *éléments*, 1973-... (1973-76; 1979-85; 1995-2002; 2006-07)	Front	BNF
			Programmatic books & conference proceedings issued by GRECE's publishing houses: Éditions Copernic & Éditions du Labyrinthe	Front	BNF
			Theoretical magazine: *Études et Recherches*, 1974-89 (1974-77; 1983-85)	Back	BNF
			Specialised theoretical magazine: *Krisis*, 1988-... (1988-2013, nr. 38/2012 on education)	Back	BNF
**Groupe d'études pour une nouvelle éducation (1976-...)**	Think tank	RR, 2nd	Monthly review: *Nouvelle éducation*, 1976-82 (1976-82)	Front	BNF
**Club de l'Horloge (1974-...)**	Think tank	RR, 2nd	Books & proceedings: Didier Maupas & le CdH, 1983, *L'école en accusation*; CdH, 1989 *Un nouveau printemps pour l'éducation*; CdH, 1989, *Identité et croissance de l'homme*	Front	BNF

Table 3: Organisations & sources for Italy – parties (a)

Org.	Type	Strand, Wave	Sources description: *title*, dates of publication (dates analysed)	Reach	Location
**Movimento Sociale Italiano, Alleanza Nazionale (1946-2009)**	Party	RR, 1st	Daily newspaper: *Il Secolo d'Italia* 1952-... (1967; 1971; 1974; 1985; 1996-7)	Front	BNC
			Magazines: *Cultura di destra* 1974-76; (1975); *Il Borghese* 1950-93; 1994-... (1998)	Front	BNC
			Party & election manifestoes 1948-2009 (1948-2009)	Front	CMP & FUS: fondo MSI, serie 2, busta 19, fasc. 52
			Speeches: searchable online	Front	archivio.camera.it
			Parliamentary activity: some MPs have published books on their school-related legislative action: N. Tripodi, 1962, *La scuola e l'uomo*; I. Giugni Lattari, 1968, *Da maggio a maggio*; MSI, 1980, *Una politica per la scuola*; F. Aloi, 1985, *Riforma della scuola*; F. Aloi, 2001, *La scuola in Parlamento*; Tripodi also collected related documents in his personal archive (Tripodi)	Front	BNC; FUS: fondo Tripodi, busta 21, fasc. 69
			Conference proceeding & educational events: MSI, 1967, *Scuola di partito*; Almirante et al., 1974, *Salvare la scuola dal comunismo*; MSI-DN, 1974, *Atti della 1a assemblea nazionale corporativa*	Back	BNC
			Newsletters & smaller reviews: various & changing titles	Back	FUS: fondo MSI, serie 1, busta 4, fasc. 12; fondo MSI, serie 4, busta 21, fasc. 63
			Proceedings of national congresses (1950-2000s) & summaries of party activity (1960-63)	Back	FUS: fondo MSI, serie 1, busta 6, fasc. 19-22; fondo MSI, serie 2, busta 19, fasc. 52-8
**Ufficio Scuola**	Party-org. MSI-school	RR, 1st	Books issued by official publishing house (Gnomes) & leadership: A. Fede, 1974, (*Per una politica dell'educazione*; A. Fede, 1981, *Problemi politici dell'educazione*; P. Siena, 1983, *Feticci dell'educazione contemporanea*; P. Siena, 1984, *Scuola del malessere*	Front	BNC
			Communication re. Consulta nazionale della scuola	Back	FUS: fondo Arani, serie 3, busta 13, fasc. 18
			Communication with local sections and unions	Back	FUS: fondo provincia Latina
**Fronte Universitario d'Azione Nazionale, Giovane Italia, Fare Fronte**	Party-org. MSI-stud.	ER/RR, 1st	Several (scattered) magazines of local & national committees: e.g., *All'Orizzonte* (1986-87)	Front	BNC
			Archival documents: newsletters of provincial committees (1960s, 1980s); fliers; proceedings of national conferences (1963; 1988/9); guidelines for activists (1960s)	Back	FUS: fondo MSI, serie 2, busta 19, fasc. 58; fondo MSI, serie 4, busta 21, fasc. 61

Table 4: Organisations & sources for Italy – parties (b)

Organisation	Type	Strand, Wave	Sources description: *title*, dates of publication (dates analysed)	Reach	Location
**Fronte della Gioventù**	Party-org. MSI-youth	RR, 1st	Several (scattered) magazines of local and national committees: *Imperium* 1950; 1954 (1950; 1954); *La Sfida* 1960-? (1960-63; 1975); *Lotta nazionale* 1972 (1972); *Spina nel fianco* (1996-97)	Back	BNC
			Archival documents: newsletters (1955-90)	Back	FUS: fondo MSI, serie 1, busta 1, fasc. 3; fondo MSI, serie 4, busta 21, fasc. 61
**Comitato Nazionale Genitori**	Party-org. MSI-parents	RR, 1st	Archival documents: communication with party leadership & local committees, organisation of elections, press-reviews	Back	FUS: fondo Arani, serie 3, busta 13, fasc. 23-27, 30-33
**Cisnal Scuola (1959-?)**	Party-org. MSI-teachers	RR, 1st	Weekly magazine: *La scuola nazionale* 1969-84 (1969-84)	Back	FUS
**Democrazia nazionale (1977-79)**	Party	RR, 2nd	Review: *Il Borghese* 1950-93; 1994-... (1977, when semi-official DN-organ); *Democrazia Nazionale* 1977 (1977)	Front	BNC

Table 5: Organisations & sources for Italy – think tanks & societal organisations

Organisation	Type	Strand, Wave	Sources description: *title*, dates of publication (dates analysed)	Reach	Location
**Istituto Nazionale Studi Politici ed Economici (1958-?)**	Think tank	RR, 1st	Archival documents: preparation of 1960 conference on The problems of Italian schooling	Back	FUS: fondo MSI, serie 2, busta 19, fasc. 55
			Proceedings of 1960 conference: Inspe, 1960, *Problemi della scuola italiana*	Back	BNC
**Sindacato Sociale Scuola (1977-...)**	Teachers' org.	RR, 2nd	Monthly magazine: *Scuola e Lavoro* 1969-2013 (1969-2013); commemoration volume: SSS, 2018, *Cinquant'anni di impegno politico controcorrente*	Front	FUS & online federazioneitalianascuola.it
			Communication & proceedings collected by SSS section of the Latina Province in Lazio (1970s)	Back	FUS: fondo SSS Latina
**Associazione Nazionale Famiglie Italiane (1978-...)**	Parents org.	RR, 2nd	Archival documents: statutes, communication with party leadership, press review	Back	FUS: fondo Arani, serie 3, busta 13, fasc. 22
**Eowyn** (?)	Cultural org. ♀	RR, 2nd	Magazine for women: *Eowyn* 1980-82 (1980-82)	Front	BNC
**Gruppo dell'Orologio (1960s-70s)**	Think tank	RR, 2nd	Monthly review: *L'Orologio* (1966; 1970; 1972)	Front	BNC
**Intervento (1972-90s)**	Think tank	RR, 2nd	Monthly magazine: *L'intervento* 1972-98 (1986-87)	Front	BNC
**La Destra (1970s)**	Think tank	RR, 2nd	Monthly magazine: *LaDestra* 1971-76 (1971-76)	Front	BNC
**Nuova destra (1979-...)**	Think tank	RR, 2nd	Monthly magazines: *La voce della fogna* 1978-83 (1978-83); *Elementi* 1978-94 (1978-79)	Front	BNC

Table 6: Organisations & sources for Germany – parties

Organisation	Type	Strand, Wave	Sources description: *title*, dates of publication (dates analysed)	Reach	Location
**National-demokratische Partei Deutschlands (1964-...)**	Party	RR, 2nd	Weekly newspapers: *NPD-Kurier* 1968-70 (1968-70); *Deutscher Kurier* 1970-75 (1971-74); *Landesspiegel* (Nordrhein-Westfahlen) 1981-89 (1989)	Front	DNB
			Manifestoes (national, 1964-2019)	Front	Party newspapers, MP, polidoc
**Die Republikaner (1983-...)**	Party	RR, 2nd	Monthly newspaper: *Der Republikaner* 1984-97 (1989; 1996-99)	Front	DNB
			Manifestoes (1989-2005, national & Baden-Württemberg)	Front	Party newspaper, MP, polidoc
**Deutsche Volksunion (1971-2011)**	Party	RR, 2nd	Manifestoes (national 1998-2003)	Front	polidoc

Table 7: Organisations & sources for Germany – think tanks & societal organisations

Organisation	Type	Strand, Wave	Sources description: *title*, dates of publication (dates analysed)	Reach	Location
**Aktion Neue Rechte (1972-...)**	Think tank	RR, 2nd	Manifesto: ANR, 1972, *Manifest einer europäischen Bewegung*	Front	www.endstation-rechts.de
**Neue Rechte: Criticón (1970-...)**	Think tank	RR, 2nd	Trimestral magazine: *Critión* 1970-2005 (1970-1998, before neoliberal turn)	Front	DNB
**Neue Rechte: Junge Freiheit (1986-...)**	Student org. & think tank	RR, 3rd	Monthly magazine: *Junge Freiheit* 1986-... (1986-2002)	Front	DNB & jungefreiheit.de
**Neue Rechte: Deutsches Kolleg (1994-...)**	Educational org. (associated to Junge Freih.)	RR, 3rd	Selected educational material accessible online 1985-2016	Front	brd-ende.com
**Neure Rechte: Institut für Staatspolitik (2000-....)**	Think tank	RR, 3rd	Monthly magazine: *Sezession* 2003-... (2003-2010)	Front	DNB & sezession.de
			Books on education: IfS, 2001, *Nationale Identität*; IfS, 2008, *Die Bueb-Debatte*; IfS, 2013, *Schulkollaps*; IfS, 2014, *Vom Heimatdienst zur politischen Propaganda*	Front	DNB & staatspolitik.de
**Neue Rechte**	Think tank	RR, 3rd	Books on schooling & education issued by publishing houses Antaios & Ares: G. Kaltenbrunner, 1984/2008, *Elite, Erziehung für den Ernstfall*; C. von Schrenck-Notzing, C., 1965/2015, *Charakter-Wäsche. Die Re-education der Deutschen und ihre bleibenden Auswirkungen*; C. Sommerfeld, 2019, *Wir erziehen*	Front	DNB

References

Abou-Chadi, T. & Krause, W. (2019). The causal effect of radical right success and mainstream parties' policy positions. *British Journal of Political Science*, *forth.*.

Albanese, M. & Del Hierro, P. (2014). A transational network: the contact between Fascist elements in Spain and Italy. *Politics, Religion & Ideology*, *15*(1), 82–102.

Almeida, D. (2019). Cultural retaliation: the cultural policies of the 'new' Front National. *International Journal of Cultural Policy*, *3*(25), 269–281.

Art, D. (2012). *Inside the radical right.* Cambridge, MA: Cambridge University Press.

Backes, U. (2018). The radical right in Germany, Austria, and Switzerland. In J. Rydgren (Ed.), *The oxford handbook of the radical right* (pp. 452–477). Oxford: Oxford University Press.

Bale, J. M. (2017). *The darkest side of politics, Vol. 1.* London: Routledge.

Bar-On, T. (2007). *Where have all the fascists gone?* London: Routledge.

Benoit, K. R., Bräuninger, T. & Debus, M. (2009). Challenges for estimating policy preferences: announcing an open access archive of political documents. *German Politics*, *18*(3), 440–453.

Birenbaum, G. (1992a). Le Front national à l'Assemblée (1986-1988). *Politix*, *5*(20), 99–118.

Birenbaum, G. (1992b). *Le Front National en politique.* Paris: Balland.

Blee, K. M. & Creasap, K. A. (2010). Conservative and right-wing movements. *Annual Review of Sociology*, *36*, 269–286.

Bozzi Sentieri, M. (2007). *Dal neofascismo alla nuova destra, le riviste 1944-1994.* Roma: Nuove Idee.

Brauner-Orthen, A. (2001). *Die Neue Rechte in Deutschland.* Opladen: Leske + Budrich.

Camus, J.-Y. (2015). Le Front National et la Nouvelle Droite. In S. Crépon (Ed.), *Les faux-semblants du front national* (pp. 97–120). Paris: Sciences Po.

Capra Casadio, M. (2014). The new right and metapolitics in France and Italy. *Journal for the Study of Radicalism*, *8*(1), 45–86.

Carter, E. (2005). *The extreme right in Western Europe: success or failure?* Manchester: Manchester University Press.

Carter, E. (2018). Right-wing extremism/radicalism: reconstructing the concept. *Journal of Political Ideologies*, *23*(2), 157–182.

Castelli Gattinara, P. & Pirro, A. L. (2019). The far right as social movement. *European Societies*, *21*(4), 447–462.

Copsey, N. (2018). The radical right and fascism. In J. Rydgren (Ed.), *The oxford handbook of the radical right* (pp. 106–122). Oxford: Oxford University Press.

Ferraresi, F. (Ed.). (1984). *La destra radicale.* Milano: Feltrinelli.

Froio, C., Castelli Gattinara, P., Bulli, G. & Albanese, M. (2020). *CasaPound Italia: contemporary extreme-right politics.* London: Routledge.

Funke, H. (2009). Rechtsextreme Ideologien, strategische Orientierungen und Gewalt. In S. Braun, A. Geisler & M. Gerster (Eds.), *Strategien der extremen rechten* (pp. 21–44). Wiesbaden: Springer VS.

Gerring, J. (2007). *Case study research. Principles and practices.* Cambridge: Cambridge University Press.

Gress, F., Jaschke, H.-G. & Schönekäs, K. (1990). *Neue Rechte und Rechtsextremismus in Europa.* Wiesbaden: Springer.

Griffin, R. (1996). The 'post-Fascism' of the Alleanza Nazionale: a case study in ideological morpholgy. *Journal of Political Ideologies*, *1*(2), 123–145.

Griffin, R. (2000). Interregnum or endgame? The radical right in the 'post-fascist' era. *Journal of Political Ideologies*, *5*(2), 163–178.

Gross, M. & Debus, M. (2018). Does EU regional policy increase parties' support for European integration? *West European Politics*, *41*(3), 594–614.

Ignazi, P. (1998). *Il polo escluso. Profilo storico del Movimento Sociale Italiano.* Bologna: Il Mulino.

Kitschelt, H. (1995). *The radical right in Western Europe: a comparative analysis.* Ann Arbor, MI: University of Michigan Press.

Krause, W. & Giebler, H. (2019). Shifting welfare policy positions: the impact of radical right populist party success beyond migration politics. *Representation*, *forth.*. doi: 10.1017/S0007123418000029

Mair, P. & Mudde, C. (1998). The party family and its study. *Annual Review of Political Science*, *1*(1), 211–229.

Mammone, A. (2015). *Transnational Neofascism in France and Italy.* Cambridge, MA: Cambridge University Press.

Mammone, A., Godin, E. & Jenkins, B. (Eds.). (2012). *Mapping the extreme right in contemporary Europe.* London: Routledge.

Mayer, N. (2018). The radical right in France. In J. Rydgren (Ed.), *The oxford handbook of the radical right* (pp. 433–452). Oxford: Oxford University Press.

Minkenberg, M. (1992). The New Right in Germany. *European Journal of Political Research*, *22*(1), 55–81.

Mudde, C. (2000). *The ideology of the extreme right.* Manchester: Manchester University Press.

Mudde, C. (2007). *Populist radical right parties in Europe.* Cambridge, MA: Cambridge University Press.

Mudde, C. (2010). The populist radical right: a pathological normalcy. *West European Politics*, *33*(6), 1167–1186.

Mudde, C. (2019). *The far right today.* Cambridge: Polity Press.

Mudde, C. & Rovira Kaltwasser, C. (2015). Vox populi or vox masculini? Populism and gender in Northern Europe and South America. *Patterns of Prejudice*, *49*(1/2), 16–36.

Norris, P. & Ingelhart, R. (2019). *Cultural backlash: Trump, Brexit and authoritarian populism.* New York: Cambridge University Press.

Pirro, A. L. P. (2018). The polyvalent populism of the 5 Star Movement. *Journal of Contemporary European Studies*, *26*(4), 443–458.

Rydgren, J. (2005). Is extreme right-wing populism contagious? Explaining the emergence of a new party family. *European Journal of Political Research*, *44*(3), 413–437.

Seidel, G. (1986). Culture, nation and "race" in the British and French New Right. In R. Levitas (Ed.), *The ideology of the new right* (pp. 107–135). Cambridge: Polity Press.

Snow, D. A. (2004). Framing processes, ideology, and discursive fields. In D. A. Snow, S. A. Soule & H. Kriesi (Eds.), *The blackwell companion to social movements* (pp. 380–412). London: Blackwell Publishing.

Tarchi, M. (2003). The political culture of the Alleanza nazionale: An analysis of the party's programmatic documents (1995-2002). *Journal of Modern Italian Studies*, *8*(2), 135–181.

Veugelers, J. (2011). Dissenting families and social movement abeyance: the transmission of neo-fascist frames in postwar Italy. *The British Journal of Sociology*, *62*(2), 241–261.

Veugelers, J. & Menard, G. (2018). The non-party sector of the radical right. In J. Rydgren (Ed.), *The oxford handbook of the radical right* (pp. 286–304). Oxford: Oxford University Press.

Volkens, A., Lehmann, P., Matthieß, T., Merz, N., Regel, S. & Weßels, B. (2017). *The Manifesto Data Collection. Manifesto Project (MRG/CMP/MARPOR). Version 2017b.* Berlin: Wissenschaftszentrum Berlin für Sozialforschung (WZB).

von Beyme, K. (1988). Right-wing extremism in post-war Europe. *West European Politics*, *11*(2), 1–18.

Zaslove, A. (2009). The populist radical right: Ideology, party families and core principles. *Political Studies Review*, *7*(3), 309–318.
